# Wheat growth model capturing growth-defense trade-off

**DOI:** 10.3389/fpls.2026.1763868

**Published:** 2026-03-12

**Authors:** Pauline Dusfour-Castan, Gerhard Buck-Sorlin, Patrice Loisel, Bénédicte Fontez, Elsa Ballini

**Affiliations:** 1 Mathématiques, Informatique et STatistique pour l'Environnement et l'Agronomie (MISTEA), University of Montpellier, Institut National de la Recherche Agronomique (INRAE), Institut Agro, Montpellier, France; 2 Institut de Recherche en Horticulture et Semences (IRHS), Institut National de la Recherche Agronomique (INRAE), Institut Agro, University of Angers, Beaucouzé, France; 3 Plant Health Institute Montpellier (PHIM) Plant Health Institute, University of Montpellier, Centre de coopération Internationale en Recherche Agronomique pour le Développement (CIRAD), Institut National de la Recherche Agronomique (INRAE), Institut de Recherche pour le Développement (IRD), Institut Agro, Montpellier, France

**Keywords:** functional-structural plant modeling, growth-defense trade-off, hormones, plant growth model, source-sink relationship, stress, wheat

## Abstract

Improving crop productivity in agroecological systems subject to multiple abiotic and biotic stresses requires a comprehensive integration of physiological mechanisms into plant growth models. In this article, we analyze the structure, components and limitations of current process-based models (PBMs) and Functional-Structural Plant Models (FSPMs) used to simulate wheat (*Triticum* spp.) growth. Although these models are well adapted to represent light interception, carbon assimilation and biomass allocation, they remain mostly oriented toward yield or growth prediction and usually neglect biotic and abiotic stress factors, which are crucial under agricultural conditions. In this article, we review the main physiological concepts of growth, including photosynthesis, nitrogen uptake, source-sink relationships and respiration costs, with an emphasis on resource allocation trade-offs. These trade-offs, particularly between growth and defense, are rarely explicitly integrated into current modeling frameworks, despite their decisive role on yield and growth under stresses. To fill these gaps, we propose a conceptual model that explicitly integrates physiological trade-offs between growth and defense, as well as hormonal signaling networks. By adopting a more explanatory and integrative approach, this work aims to improve the ability of models to facilitate the transition towards a stronger integration of agroecological principles.

## 1 Introduction

The agricultural sector is confronted with the urgent need to increase food production to meet the challenge of a constantly increasing global population while addressing climate change and minimizing environmental impacts. This requires sustainable farming practices that optimize yield while reducing resource usage and carbon emissions. Against this backdrop, farming practices are evolving towards more complex cropping systems based on improved understanding of plant physiology, including varietal associations, optimized plant-soil interactions, and integrated management of both abiotic and biotic stresses. Historically, crop and plant growth models have been designed as predictive tools for estimating yields under simplified conditions, or those considered to be optimal (Guarin and Asseng, 2017). Now, faced with these new challenges, it is worth questioning whether these models can capture the diversity and complexity of agroecological farming systems. To better support experimental design and virtually test the impact of different agroecological practices, we require models that integrate both predictive and explanatory approaches. While current models have made significant progress in considering environmental factors, they remain primarily oriented toward yield prediction under given conditions. In contrast, explanatory models aim to capture the underlying biological regulation and system behaviour, i.e. the physiological and molecular mechanisms that govern plant responses to environmental variation. This mechanistic understanding is essential for making informed decisions about sustainable farming practices and developing effective strategies to minimize environmental impact.

**Figure 1:**
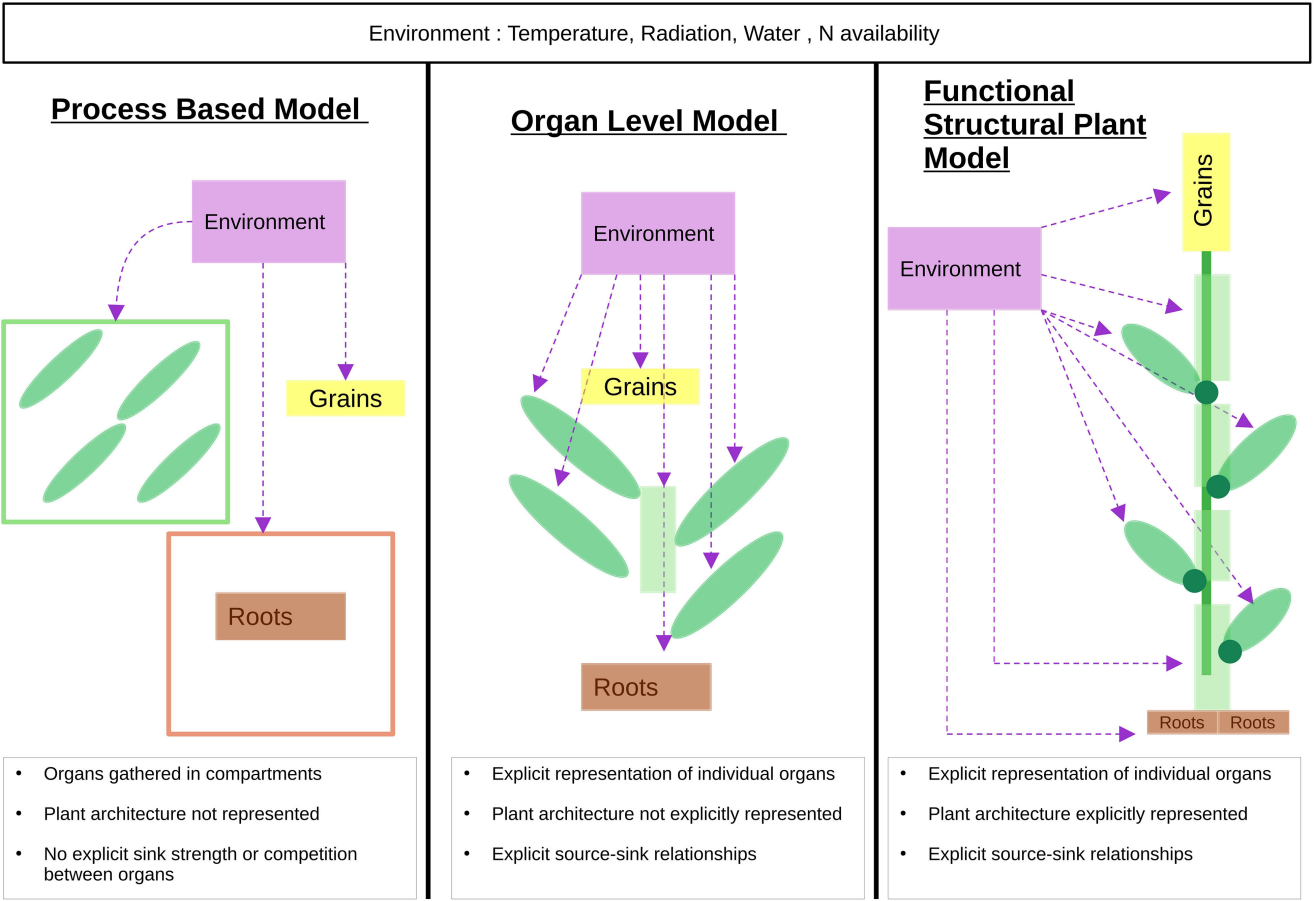
Schematic comparison between Process-Based Models (PBMs), intermediate organ-level representation, and Functional-Structural Plant Models (FSPMs). Dashed arrows indicate environmental influences on organs or compartments. The representation of plant organs and their spatial arrangement reflects the degree of architectural detail included in each modeling framework. Left panel (PBM): Organs are represented as aggregated compartments. Plant growth is represented through mass balances at the compartment scale, without explicit representation of three-dimensional plant architecture. Physiological processes operate at the compartment scale, and assimilates are typically distributed among compartments through common pools. Source-sink relationships are represented in a simplified and non-spatial manner. Middle panel (Organ-level model): Individual organs are explicitly distinguished, allowing representation of organ interactions and source-sink relationships. However, plant architecture remains implicit and the 3D structure is neglecting. Right panel (FSPM): Organs are represented individually with their topology, geometry and spatial organization. Environmental factors influence each organ locally, allowing representation of microclimate effects. Physiological processes are explicitly coupled with plant architecture, enabling explicit source-sink relationships and inter-organ resource flows.

Wheat *(Triticum* spp.*)*, which accounts for around a fifth of the calories in the human diet (Gooding, 2023), is one of the most extensively studied crops. The first wheat growth models were developed in a multidisciplinary manner to enable scientists to test their growth hypotheses (Day and Atkin, 2013). In particular, the work of de Wit (1978) marked an important turning point in the field of agronomical modeling with the creation of the SUCROS model (Van Keulen and Wolf, 1986), which simulates plant growth as a function of light interception and utilization efficiency. Subsequently, in the 1970s and 80s, more specific models were developed, such as CERES-Wheat (Ritchie and Otter, 1985) and ARCWHEAT (Weir et al., 1984), that further integrated plant physiological processes such as photosynthesis and respiration. While these approaches could be classified under the category of Process-Based Models (PBM), since the 1990s, we have seen a diversification of models with the development of new approaches, such as Functional-Structural Plant Models (FSPM), which integrate complementary aspects to describe and predict plant growth in interaction with its environment and with a strong consideration of plant architecture (**Figure 1**). The FSPM paradigm considers the plant as an assembly of elementary units, and takes into account both the physiology of a plant and its environment (Vos et al., 2010; Buck-Sorlin, 2013). Both PBM and FSPM models are generally organized into several distinct modules, each one of which represents a specific organ or physiological function of the plant or its environment, which interact with each other to realistically simulate the complex processes involved in growth and development. In the case of wheat, these multi-module models include (i) phenology, which describes the different phases of wheat development as a function of temperature and sometimes photoperiod, (ii) biomass accumulation as a function of light interception and photosynthesis, (iii) water and nitrogen balances as a function of soil water dynamics and nitrogen absorption by roots, and (iv) resource allocation as a function of the requirements of each organ. In addition, several studies have assessed the effects of climate change on growth and yield, considering in particular the impact of temperature variations (Asseng et al., 2011; Liu et al., 2016), water stress (Yang et al., 2023; Zhao et al., 2020) and genetic aspects (Khan and Mohammad, 2018) in order to integrate them in the respective models.

Indeed, climate variability not only imposes direct abiotic constraints on plant growth but also alters the dynamics of biotic interactions, notably by modifying pathogen pressure and pest development cycles. To cope with such combined stresses, plants deploy multiple defense strategies, including adjustments to phenological patterns, modifications of physiological processes, increased synthesis of protective metabolites and recruitments of auxiliaries (Medda et al., 2022). These defense responses involve both metabolic pathways, such as the production of secondary compounds including phenolics, alkaloids, and terpenoids, and hormonal signaling networks that coordinate stress perception and response. However, physiological defense mechanisms and varietal resistances are often overlooked in current models, along with the trade-offs associated with resource allocation between growth and defense. Critically, the activation of defense mechanisms requires significant reallocation of carbon and nitrogen resources away from growth processes, imposing metabolic and energetic costs that directly impact plant performance (Sestari and Campos, 2022). By explicitly incorporating these physiological defense mechanisms, their resource costs, and the resulting growth-defense trade-off into models, researchers can better understand plant-environment interactions under stress and develop strategies to minimize the impacts of biotic stresses on crop yields while supporting more informed decisions about sustainable farming practices.

In this context, the aim of the present work is first to outline the different approaches used to model wheat growth, highlighting their strengths and limitations in the face of current challenges. Particular attention will then be paid to the consideration of biotic stresses whose integration remains limited today. Finally, we will develop a plant-level conceptual model that explicitly captures physiological trade-offs in resource allocation to growth, defense, and reproduction, and lays out how these balances shift in response to fluctuating environmental conditions, providing a more explanatory and realistic framework for wheat performance under complex agroecological contexts.

## 2 What components are needed to predict growth in agroecological systems?

In a context of climate change and the quest for agricultural productivity, the need for high-performance growth models has become paramount. PBMs and FSPMs have emerged as two complementary approaches to plant and crop modeling (**Figure 1**).

In these two modeling paradigms, the various physiological processes of the plant are simulated in interaction with its environment, and the plant is considered as an assembly of modules associated with specific functions such as photosynthesis, respiration or resource uptake. This modular approach makes it possible to precisely describe interactions between organs (e.g., nutrient flow) and with the environment (e.g., light interception). PBMs are mainly used to predict crop yield, illustrating development and growth in terms of mass variables per unit leaf area (Vos et al., 2010; Vos and Heuvelink, 2006). However, PBMs are based on the scale of compartments and do not consider the plant’s 3D architecture, which limits their modeling capacity. To overcome this problem, FSPMs aim to simultaneously represent the plant’s physiological processes and its 3D structure over time (Vos et al., 2010; Buck-Sorlin, 2013). They assume that plants respond to their environment not only by adjusting their physiological functions but also by modifying their structure. Their modular approach makes it possible to represent the plant by considering the topology and geometry of each organ, as well as its exposure to a specific microclimate (“phylloclimate” according to Chelle (2005)). Based on the notion of organ topology and geometry, this structural approach also allows representation of materials and information flows between organs, giving access to a more precise model of plant functioning.

In the specific context of wheat, models such as GreenLab (De Reffye et al., 1997), CN-Wheat (Barillot et al., 2016), NEMA (Bertheloot et al., 2011), WHEAMM (Gawinowski et al., 2023), and ADEL-Wheat (Fournier et al., 2003; Evers et al., 2006, 2010) have collectively advanced modeling capabilities by integrating carbon-nitrogen interactions, organ-level resource allocation, and architectural dynamics. These advances are particularly relevant for agroecological systems, where heterogeneous canopies, intercropping, and varietal mixtures create complex light environments and competitive interactions that cannot be adequately captured by compartmental models alone. FSPMs, with their explicit representation of 3D architecture and local microclimates, are especially well-suited for simulating such spatial heterogeneity and to explore how canopy structure influences resource capture and biotic interactions.

Whatever the modeling approach adopted, the representation of plant growth requires consideration of certain fundamental elements. These include phenological development, which conditions the evolution of plants over time, and the processes involved in the acquisition of essential resources, as well as their allocation to the various organs. These basic mechanisms ensure a certain realism when describing plant growth and understanding how it is modulated by the environment.

### 2.1 Core physiological mechanisms and their integration in plant growth models

**Figure 2:**
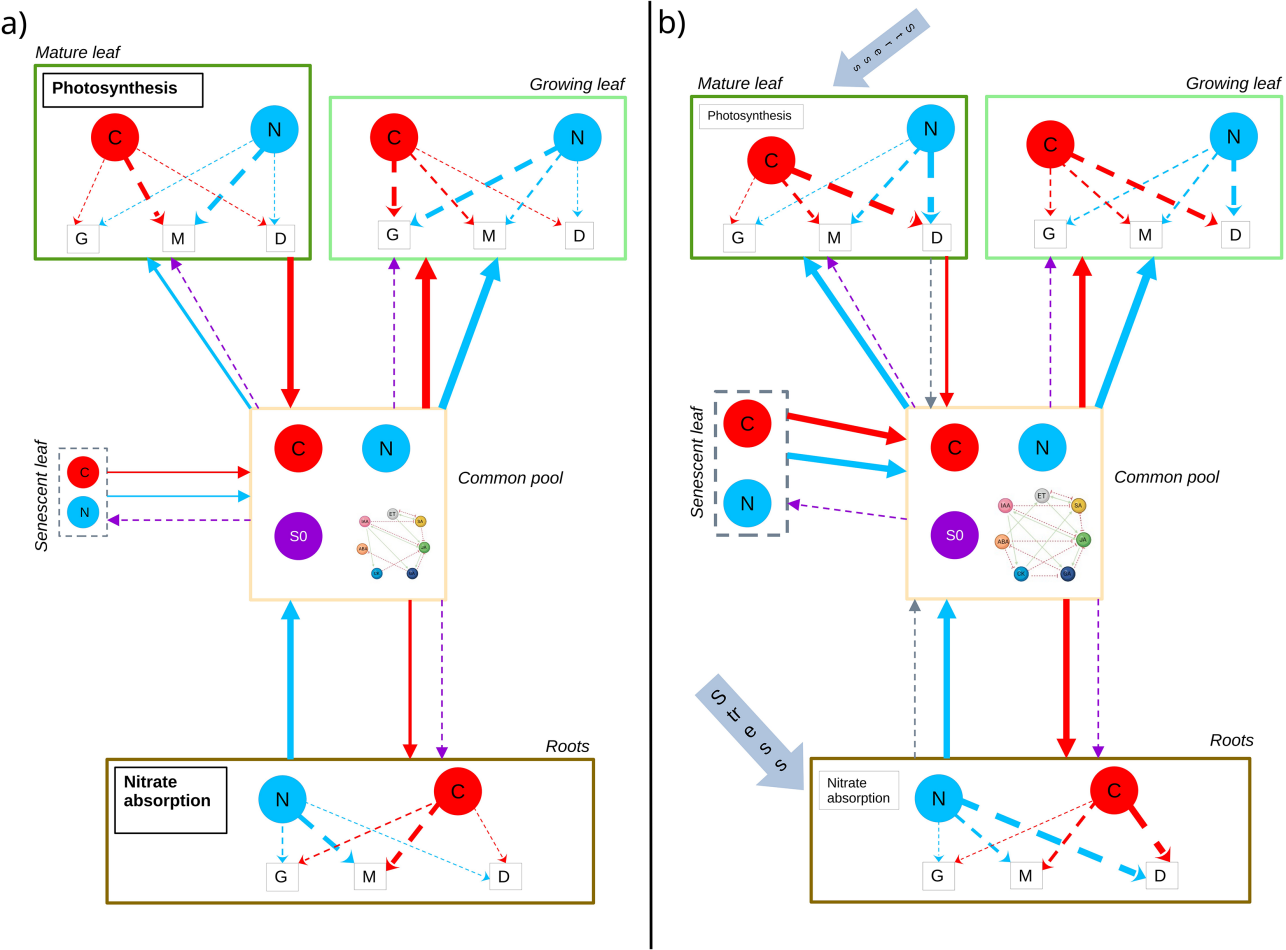
Schematic representation of carbon (C) and nitrogen (N) assimilation and allocation in a wheat under no-stress and stress conditions. Solid red and blue arrows represent inter-organ fluxes of carbon (red) and nitrogen (blue) through the common pool, reflecting source-sink flows between organs. Dashed blue and red arrows represent the internal allocation between growth (G), maintenance (M), and defense (D) of nitrogen and carbon within each organ. Dashed purple arrows represent the transmission of global plant status information (signal S0) from the common pool toward organs. This signal integrates information about the overall physiological state of the plant and modulates allocation rules and sink strengths. Under stress conditions, dashed grey arrows represent stress signals emitted by organs toward the common pool, updating the global plant status and consequently modifies the signal S0 governing source-sink relationships and allocation rules at the organ level. The hormonal network coupled to signal S0 represents the plant’s “decision system”, modulating source-sink relationships and organ-level allocation rules in response to environmental conditions and information sent from organs. **(a)** Mature leaves perform photosynthesis and roots absorb nitrates. Carbon and nitrogen are then transferred to the common pool and redistributed to growing organs according to baseline source-sink relationships. Allocation primarily supports growth and maintenance, with minimal investment in defense. Source capacities are fully operational, and senescent leaves contribute to remobilization without stress-induced acceleration. **(b)** Stress conditions. Under biotic or abiotic stress, organs emit local stress signals that alter allocation priorities. A larger fraction of carbon and nitrogen is redirected toward defense and maintenance, reducing the amount available for growth. Photosynthetic capacity and/or nitrate uptake may decline, decreasing source strength. Stress-induced senescence can be accelerated, promoting remobilization of resources to sustain critical functions. These modifications reflect a dynamic reorganization of source-sink relationships between organs in response to stress.

One of the main physiological mechanisms underlying plant growth is photosynthesis (**Figure 2a**). This bioenergetic process enables the plant to synthesize organic matter by transforming light energy into chemical energy. For the sake of simplicity, we will assume that the carbon assimilated by the plant comes essentially from this process, and that dry matter production depends on the rate of photosynthesis. A detailed representation of this mechanism is essential to accurately quantify plant growth. A wide variety of photosynthesis models exist, depending on the spatial scale considered, the research question, and the degree of precision with which the underlying mechanisms are represented. Most models include the FvCB model of Farquhar et al. (1980), which describes the main biochemical and photochemical processes involved in photosynthesis. This model is used to estimate the rate of *CO*_2_ assimilation as a function of leaf temperature, photosynthetically active radiation (PAR), and *CO*_2_ and *O*_2_ concentrations at the carboxylation sites. Environmental variables such as relative humidity are incorporated indirectly through their effects on stomatal conductance. A set of equations and parameter definitions used in this formulation are provided in **Supplementary Table 1**. This model is based on the assumption that assimilation is constrained either by the flow of photosynthetic electrons required to regenerate RuBP, or by the quantity and state of activation of Rubisco, or else by the utilization of triose phosphates. Often coupled with the BWB (Ball-Woodrow-Berry) stomatal conductance model of Ball et al. (1987), the combination of these two models, though largely empirical, provides a better representation of gas exchange and plant responses to environmental conditions. The transpiration rate is generally calculated using diffusion equations applied to water vapor exchanges, and the temperature dependence of the parameters is defined using Arrhenius functions, taking a reference temperature of 25 °C.

In the context of growth modeling, nitrogen content is a determining factor in the activity of certain processes (Jeuffroy et al., 2002). For example, nitrogen content directly influences the photosynthetic capacity of leaves by regulating the synthesis of proteins required for carbon fixation, thus influencing the carboxylation rate and electron transport (Müller et al., 2005). In order to accurately represent physiological processes related to nitrogen, it is essential to consider the mechanisms regulating its uptake by the plant. Nitrogen, mainly absorbed by roots (**Figure 2a**) in the form of nitrates, is captured by specific transport systems: (i) the high-affinity system (HATS), and (ii) the low-affinity system (LATS), enabling the plant to adjust its uptake according to soil nitrate availability (Demotes-Mainard and Jeuffroy, 2004; Ben Slimane, 2010). The relationship between soil nitrate concentration and nitrogen uptake is generally represented by a Michaelis-Menten type function for the HATS transport system, describing a progressive saturation of the uptake rate as nitrate availability increases, and by a linear function for the LATS system, active mainly at higher concentrations.

Once absorbed and assimilated by the plant, nitrogen plays an active role in regulating physiological processes, including respiration. This process, just as essential to be represented in growth models, is involved in the consumption part of the carbon assimilated during photosynthesis, and has a direct influence on the plant’s energy balance and growth. Respiration takes place both day and night, and in particular enables the production of the energy and reducing power required for various physiological functions (Plaxton and Podestá, 2006). This highly regulated metabolic mechanism can be adjusted to environmental conditions, enabling the plant to adapt to stressful situations or variations in resources. In most models, respiration is divided into two components, (i) a growth component, and (ii) a maintenance component. Growth respiration is involved in the processes required for the biosynthesis of structural components, while maintenance respiration refers to the processes involved in maintaining structures already in place and in protein turnover (Boote et al., 2013). The daily amount of *CO*_2_ produced per unit leaf area during respiration is then considered to be proportional to the rate of photosynthesis and plant mass (Amthor, 2000; Thornley, 2011). Noting that the costs associated with each process can vary independently in response to environmental changes, Thornley and Cannell (2000) proposed a second approach called the general paradigm, which consists of considering individually the relationships between each biochemical process and its respiratory costs. The daily amount of *CO*_2_ produced per unit leaf area is then the sum of the activity rate of the processes multiplied by their metabolic cost. Among the processes taken into account in this calculation are mechanisms linked to the growth of each organ, phloem charge rate, nitrate uptake, reduction and fixation, ammonia and mineral uptake, as well as a residual share that includes respiration linked to maintenance processes.

### 2.2 Beyond common pools: mechanistic coupling of carbon and nitrogen flows in plant source-sink dynamics

In addition to the physiological mechanisms described above, plant growth is highly dependent on the internal exchange of matter between different organs. These exchanges are governed by source-sink relationships, an essential concept for understanding how resources are distributed within the plant according to the specific needs of each organ. Plant growth and development depend on efficient resource management, with elements distributed according to a hierarchical organization involving source organs and sink organs. A “source” organ is one that produces or releases a resource required for growth, while a “sink” organ is a consumer (White et al., 2016). Modeling source-sink relationships has become indispensable to improve the predictive and explanatory capacity of models as well as to better understand the internal dynamics of plants.

Usually, resources assimilated by the plant are considered to be stored in a common pool and distributed according to organ demand. Many molecules, such as water and various minerals, are essential for growth, but nitrogen and carbon are generally the only elements explicitly represented in source-sink relationships. Traditionally, mature leaves are thought of as carbon sources, while young leaves, roots and grains act as sinks. With regard to nitrogen, roots play the role of source, while leaves and grains take on the status of sink (White et al., 2016). However, carbon and nitrogen flows are not independent, but rather tightly coupled through stoichiometric constraints that reflect the biochemical composition of plant tissues. Most structural tissues require relatively fixed C:N ratios: for example, leaf tissue typically maintains C:N ratios between 10:1 and 15:1, while proteins have much lower C:N ratios around 3.5:1. This coupling means that carbon allocation to a growing organ cannot proceed independently of nitrogen availability, and vice versa. When nitrogen is limiting, excess carbon may be redirected to storage compounds (starch, lipids) or secondary metabolism, while under carbon limitation, nitrogen uptake and assimilation may be downregulated to avoid accumulation of toxic nitrogenous compounds.

Different modeling frameworks handle source-sink relationships with varying degrees of complexity. GreenLab is characterized by an explicit sink-source notion, though the model focuses exclusively on carbon exchanges. Resources stored in the common pool are dispatched between different organs according to demand and sink strength. An organ’s sink strength refers to its ability to compete with other organs for access to biomass (Kang et al., 2008). This strength may evolve over time, following a beta function, but remains identical for all organs belonging to the same type (e.g., leaves or grains). In contrast, CN-Wheat incorporates both carbon and nitrogen explicitly, offering a more complete vision of inter-organ interactions through coupled C-N allocation algorithms. However, even in CN-Wheat, the coupling is typically implemented through empirical allocation coefficients rather than mechanistic representations of stoichiometric constraints and tissue-specific C:N requirements. This highlights an important limitation in current modeling approaches: the lack of mechanistic coupling between carbon and nitrogen flows means that models may fail to capture how limitation of one resource cascades through the entire allocation network, particularly under stress conditions where optimal C:N ratios may shift due to defense compound synthesis or stress-induced metabolic adjustments. Moreover, this concept of source-sink relationships, essential for accurately representing plant growth, can evolve according to the plant’s physiological needs and in response to environmental variations. For example, during the vegetative phase, mature leaves act mainly as carbon sources for the growth of young leaves, while roots act as sinks. Conversely, during the reproductive phase, seeds become the dominant sinks, actively mobilizing the resources accumulated in vegetative organs. This transition is closely linked to a fundamental process: programmed senescence (Borrill et al., 2019).

In most current crop models, senescence is implemented through relatively simple functions: either fixed schedules based on thermal time or developmental stage (e.g., senescence begins at a specified number of degree-days after anthesis), or threshold-based triggers responding to single environmental variables (e.g., senescence accelerates when soil water content drops below a critical threshold). While computationally efficient, these simplifications fail to capture the dynamic nature of senescence as a regulated process that responds to multiple interacting signals including nutrient status, hormonal cues, pathogen presence, and source-sink imbalances. In reality, senescence timing and progression are modulated by complex feedback loops: for example, a strong reproductive sink can accelerate leaf senescence to mobilize nitrogen for grain filling (a phenomenon known as “monocarpic senescence”), while biotic stress can trigger localized senescence around infection sites as part of the hypersensitive response. Moreover, the rate of nutrient remobilization from senescing tissues is not constant but depends on the demand from active sinks and the plant’s overall nutrient status. By representing senescence through fixed schedules or simple thresholds, models miss these dynamic feedbacks and may misrepresent resource availability during critical growth phases, particularly under variable stress conditions. Despite its importance in resource allocation and nutrient use efficiency, senescence remains a complex process that is still poorly understood, particularly in leaves and roots (Camargo Rodriguez, 2021), and its mechanistic representation in models remains a significant challenge.

## 3 Rethinking crop growth models in an agroecological context

Growth models are essential for the development of sustainable cropping systems. However, these models can sometimes have certain shortcomings and therefore do not reflect the reality in the field. In particular, the practical implementation of these models remains challenging due to their dependence on numerous input data, that are often difficult to obtain under outdoor conditions. This lack of availability often forces certain variables to be estimated, introducing additional uncertainty that can alter the accuracy of predictions (Alderman and Stanfill, 2017; Kasampalis et al., 2018). These shortcomings also include insufficient integration of physiological processes that are highly sensitive to environmental conditions. Current PBM and FSPM frameworks remain limited with respect to their representation of stress responses and physiological trade-offs. Abiotic stresses (drought, heat, nutrient limitation) and biotic stresses (pathogens, pests) impose constraints on resource availability and trigger defense responses that compete with growth for carbon and nitrogen. Integrating these trade-offs requires models to explicitly account for: (i) stress-induced shifts in resource allocation, whereby carbon and nitrogen are diverted from structural growth to defense compound synthesis or stress mitigation pathways; (ii) competition between organs for limited resources under stress, modulated by dynamic sink strengths that reflect both developmental priorities and stress-induced demands; and (iii) hormonal signaling networks that coordinate whole-plant responses, adjusting allocation patterns in response to environmental cues. By incorporating these mechanisms, PBMs and FSPMs could transition from purely predictive tools to explanatory frameworks capable of capturing the dynamic balance between growth, defense, and maintenance under fluctuating agroecological conditions.

Many wheat physiological processes, such as leaf emergence rate, root growth or the onset of anthesis, are particularly sensitive to abiotic conditions (Boote et al., 2013). For example, severe thermal stresses have been shown to adversely affect germination, seedling emergence, flowering and anthesis (Venkatesh et al., 2022). However, current wheat growth models still neglect the effect of extreme climatic conditions such as rising temperatures, rising *CO*_2_ concentrations or prolonged drought episodes on these developmental processes. This shortcoming significantly limits their predictive capacity in the face of ongoing climate change (Liu et al., 2023). On the other hand, despite its primordial role in describing carbon and nitrogen fluxes, the root system is often neglected. Its overly simplistic modeling generally omits fundamental parameters such as rooting depth, which nonetheless contributes to resilience in the face of water stress. In addition, important characteristics such as length, diameter, surface area and root density are rarely accurately represented, limiting the ability of models to explore the soil and access resources. Another largely underestimated aspect is root exudation, which strongly influences nutrient availability and soil interactions (Fourcaud et al., 2008). A superficial modeling of the soil therefore also has a strong impact on model performance, and compromises a realistic assessment of plant-soil interactions. Structure, porosity and water retention capacity are often ignored, which distorts estimates of water uptake and physical stresses on roots. Finally, organic matter fluxes, nutrient mineralization and gas exchanges are rarely taken into account with any precision, which limits our understanding of the processes governing soil fertility and resilience to environmental stresses. Nevertheless, efforts to model root architecture and function have significantly improved the representation of root processes, notably by integrating dynamic root growth, 268 hydraulic properties, and interactions with soil structure into crop models (Lobet et al., 2011; Pagès et al., 2014; Baca Cabrera et al., 2025).

Moreover, the limitations of current models are not confined to the abiotic component of the soil-plant system. Biotic factors such as pests and diseases are still rarely incorporated into wheat growth models, despite their considerable impact on crop health and yield quality (Oerke, 2006). Although some recent studies have begun to explicitly integrate these aspects (Garin et al., 2014; Whish et al., 2015; Triki et al., 2023; Barillot et al., 2025), the underlying physiological trade-offs remain largely neglected. However, these biotic factors directly impact resource allocation within the plant, notably by inducing a redirection of resources towards defense mechanisms at the expense of growth. In addition, when plants are exposed to a combination of abiotic and biotic stresses, these allocation trade-offs become even more pronounced. In practice, the energetic costs associated with the activation of defense mechanisms are rarely explicitly represented in models, but rather implicitly integrated into a maintenance term. More specifically, this term is often used as a “catch-all” in which the costs associated with various physiological processes are hidden. According to Wendering and Nikoloski (2023), the lack of precise mechanistic representation of these costs in models limits their ability to correctly capture actual energetic and metabolic trade-offs, thus reducing their explanatory power.

Consequently, agroecological and stress conditions also challenge the fundamental assumptions underlying classical representations of source–sink relationships in growth models. The parameterization of source–sink relationships presents particular challenges under such conditions, as the assumptions underpinning standard modeling approaches may no longer hold. First, most source-sink parameters (sink strengths, potential growth allocation coefficients, remobilization rates) are calibrated under controlled, near-optimal conditions and may not hold when plants experience nutrient limitation, water stress, or biotic pressure. For instance, the beta functions commonly used to describe temporal patterns of sink strength assume consistent developmental trajectories, but these trajectories can be substantially altered by stress: heat stress can accelerate grain filling while reducing final grain weight, effectively changing both the timing and magnitude of grain sink strength. Second, the common-pool assumption, whereby all assimilated resources are immediately available for allocation to any sink, becomes problematic under stress. Experimental evidence suggests that under pathogen attack or localized stress, resources may be preferentially retained in source leaves or redirected to specific organs (e.g., roots under drought, or sites of infection under biotic stress) rather than being freely distributed according to pre-determined sink strengths. This represents a stress-induced override of normal allocation priorities that is rarely captured in current models. Third, in agroecological systems featuring intercropping, varietal mixtures, or heterogeneous canopies, individual plants or plant types may experience asynchronous stress exposure and developmental timing, leading to highly variable source-sink dynamics within a crop stand. Models that rely on uniform parameterizations across all individuals may therefore fail to represent the emergent properties of such diverse systems. Finally, the introduction of growth-defense trade-offs fundamentally alters source-sink dynamics by creating a new category of sinks, defense related processes, that compete with growth sinks for resources but are not represented in traditional source-sink frameworks. Under biotic stress, organs under attack, if they are not actively shed or otherwise destroyed, may switch from being sources (mature leaves producing photosynthate) to being net sinks (consuming resources for local defense responses), a reversal that challenges the fixed source-sink classifications used in most models. Addressing these challenges requires more flexible, mechanistic approaches to source-sink modeling that can accommodate stress induced changes in allocation priorities, represent the costs of defense as explicit sinks, and dynamically adjust parameters based on plant physiological state and environmental conditions.

In this context, the representation of resource allocation remains a central challenge in plant growth modeling. Growth, reproduction, maintenance, and defense are conditioned by the strategic distribution of a limited pool of assimilated resources among organs and functions, reflecting physiological trade-offs arising from biochemical, structural, and environmental constraints. Because these allocation flows are difficult to quantify directly in natural systems, modeling approaches have historically relied on simplifying assumptions. For example, Yang and Midmore (2005) proposed a dynamic model in which plants adjust their resource allocation to favor growing organs, depending on local nutrient status and vascular network structure. Although this model is capable of reproducing a number of specific allocation scenarios, it focuses solely on growth and neglects the costs associated with other vital functions, such as defense or storage. More recently, other approaches, such as dynamic energy balance (DEB) models, have attempted to unify allocation processes (Russo et al., 2022). Despite them offering a robust and coherent theoretical framework, DEB models have several limitations when it comes to representing plants. In particular, they struggle to integrate dynamic shifts in physiological processes and their dependence on biotic constraints. At the same time, emerging mechanistic approaches, notably that of Monson et al. (2022) have proposed the concept of coordinated resource allocation, according to which plants actively adjust their resource flows between growth and defense based on regulation networks integrating hormonal signals. This theoretical framework makes it possible to link cellular mechanisms to physiological responses observed at plant level, and opens the way to more realistic and explanatory modeling allocation strategies in plants, particularly in complex or stressful agroecological conditions.

## 4 What components should be added to take stress factors into account?

### 4.1 Dynamic resource allocation under stress: bridging defense mechanisms and growth trade-offs in plant models

Plants are constantly exposed to a variety of environmental stresses that can have a major impact on their growth and survival. This includes abiotic factors such as drought, rising temperatures and *CO*_2_ concentrations, or salinity, as well as biotic factors such as pathogens or pests. As a combat strategy, plants have developed a sophisticated set of response mechanisms that consist in the detection, limitation and neutralisation of stress factors and threats (Jones and Dangl, 2006; Bigeard et al., 2015; Du et al., 2024). These processes rely on complex hormonal signaling networks to trigger an adaptive response (Yang et al., 2015). The general assumption of the growth-defense trade-off is that the implementation of defense mechanisms requires a significant amount of resources, leading to a reduction in growth (Herms and Mattson, 1992; He et al., 2022).

As described in part 2.1, photosynthesis, nitrogen uptake, and respiration form the metabolic foundation that determines the pool of resources available (carbon and nitrogen) for allocation within the plant. Under non-limiting conditions, the carbon and nitrogen assimilated through these processes support structural growth, reproduction, and basic maintenance (**Figure 2a**). However, this resource pool is finite, and plants must continuously prioritize allocation among competing demands, a constraint that becomes particularly acute under stress. When plants are exposed to biotic stresses such as pathogen attack or herbivory, a significant portion of assimilated carbon and nitrogen is diverted away from growth-supporting processes toward the synthesis of defense compounds (**Figure 2b**) (Huot et al., 2014). These include a diversity of strategies at different scales. Defense activation at the tissue and organ levels can lead to localized cell death or accumulation of toxic molecules. The production of these defense molecules imposes substantial metabolic and energetic costs that can be equivalent to approximately 10-15% of total photosynthate under pathogen challenge, with corresponding increases in maintenance respiration to support defense-related enzyme activity (Massad et al., 2012). Moreover, the metabolic shift toward producing defense compounds (e.g., phenolics, terpenoids, alkaloids, and pathogenesis-related proteins) often reduces photosynthetic efficiency, alters nutrient distribution, while excessive defense hormones may further suppress growth pathways or plant development. At the whole-plant level, these trade-offs manifest as systemic growth retardation, modification of microbiota interactions and finally yield loss.

Plant responses to stress are organized into functional modules—semi-independent physiological processes at the tissue or organ level that can be dynamically coordinated or decoupled based on environmental cues. This modularity allows plants to adjust resource allocation between growth and defense without disrupting unrelated functions, as seen when infected tissues activate defense pathways while uninfected tissues maintain growth-oriented processes. Such compartmentalization enables fine-tuned adaptability across diverse stress conditions (Kurepa and Smalle, 2023). From a modeling perspective, integrating this modular organization into PBMs or FSPMs requires representing hormone signaling as dynamic state variables at the organ or tissue level, with explicit rules governing how local hormone concentrations influence resource allocation (**Figure 2**). These dynamics include signal delays (time lags between stress perception and hormone accumulation), feedback loops (positive or negative regulation of hormone synthesis), and hysteresis (persistence of stress responses based on prior exposure), which collectively shape realistic response patterns such as priming, where mild stress enhances responsiveness to subsequent challenges.

However, these modular responses do not fully capture the total cost of defense. Defense costs extend beyond allocation trade-offs and occur at multiple scales (Cipollini and Walters, 2014). Metabolic costs refer to the carbon and nitrogen diverted from growth to synthesize defense compounds: for example, producing one gram of phenolic compounds may require approximately 2–3 grams of glucose equivalents and can immobilize nitrogen in defense proteins rather than Rubisco. The carbon cost of synthesizing secondary metabolites may represent only a small fraction of the plant’s carbon budget, suggesting that yield reductions linked to defense are not solely due to resource diversion (Foyer et al., 2007). Energetic costs encompass the additional respiration required to fuel defense-related processes, including ATP consumption for active transport of defense compounds, energy-intensive post-translational modifications, and elevated maintenance respiration in tissues with high concentrations of short-lived defense proteins. Structural costs involve growth reductions due to resource reallocation and altered developmental programs, such as thickened cell walls that reduce leaf expansion or early senescence that shortens the photosynthetic lifespan of leaves. Finally, plant stress responses likely emerge from interactions among multiple regulatory pathways, combined with prioritization rules that allocate resources and regulatory effort to specific organs or functions depending on developmental stage and stress context (Wolinska and Berens, 2019). Two non-exclusive hypotheses describe this prioritization: the protection of young or actively growing organs, and a compartmentalized organization where individual organs or tissues exhibit partially autonomous stress responses (Wolinska and Berens, 2019).

### 4.2 The potential role of hormonal networks for integrating plant response to the environment

Although many plant responses to environmental stresses have been extensively characterized, most growth models still rely on simplified formulations in which stress factors are treated independently or through fixed correction functions. While such approaches may be adequate for describing isolated stress effects, they rapidly reach their limits under agroecological conditions, where plants are frequently exposed to multiple, simultaneous stresses. In particular, the combined occurrence of abiotic and biotic stresses challenges the assumption of additive responses. Experimental evidence shows that plant responses to stress combinations are often specific and non-additive, and cannot be inferred from responses to individual stressors alone (Suzuki et al., 2014; Khan et al., 2025). Although some modeling frameworks have begun to incorporate stress interactions, they generally remain limited to a small number of factors and rarely account for combinations involving biotic stresses (Webber et al., 2022). This gap largely reflects the complexity of the underlying regulatory mechanisms: when several stresses occur simultaneously, multiple hormonal and signaling networks are activated, often interacting in synergistic or antagonistic ways, making plant responses highly context-dependent and difficult to accommodate within current modeling paradigms (Zandalinas and Mittler, 2022; Adamik et al., 2023).

Even though many molecular mechanisms underlying the growth-defense trade-off remain to be fully elucidated, hormonal networks have emerged as central components of plant signal integration. Rather than acting in isolation, phytohormones form interconnected regulatory networks that integrate biotic and abiotic stress signals and translate them into coordinated physiological responses (**Figure 2b**). Salicylic acid (SA), jasmonic acid (JA), and ethylene (ET) are central regulators of defense against pathogens and herbivores, whereas abscisic acid (ABA) mainly controls abiotic stress responses such as drought. In contrast, auxin (IAA), gibberellins (GA), and cytokinins (CK) primarily regulate growth and development processes. Through extensive cross-talk, these hormonal pathways jointly modulate gene expression, defense activation, and growth-related processes, thereby playing a pivotal role in coordinating stress responses and overall plant performance (He et al., 2022).

This categorization, however, provides an oversimplified representation of a regulatory system that is highly context-dependent and still not fully understood. It appears that hormones like SA can sometimes be involved in development and conversely hormones like CK can be involved in defense under specific environmental conditions or at a specific genetic background. Indeed, plant genotypes can exhibit differences in signal information-flow and biochemical responses to stress (Kalapos et al., 2016). Several examples illustrate how these phytohormones complement or oppose each other to orchestrate trade-offs between growth and defense, allowing plants to adapt their responses to the context while limiting their energy costs (Bari and Jones, 2009; Huot et al., 2014). A well-documented example is the antagonistic interaction between auxin and SA signaling pathways, which illustrates how growth and defense activation can mutually constrain each other (Chan, 2022). These relationships, which remain difficult to characterize precisely, underline the complexity of modeling growth-defense trade-off and resource allocation (Aerts et al., 2021).

However, plant responses to stress do not appear to be governed by a single centralized hormonal network acting uniformly across the organism. They likely emerge from the interaction of multiple regulatory pathways, combined with additional prioritization rules whereby resources and regulatory effort are allocated preferentially to specific organs or functions depending on developmental stage and stress context (Wolinska and Berens, 2019). Two main, non-exclusive ideas have been proposed to describe this prioritization. The first emphasizes the protection of young or actively growing organs. The second points to a more compartmentalized organization, in which individual organs, or even tissues, can exhibit partially autonomous responses to stress. Together, these perspectives highlight that stress integration and plant responses are spatially structured and context-dependent, representing major challenges for their representation in whole-plant growth models.

Beyond their regulatory complexity, compound stresses also have profound consequences for internal resource allocation. Abiotic stresses such as drought or nutrient limitation directly constrain the size of the assimilate pool by reducing photosynthesis and nitrogen uptake, while biotic stresses induce defense responses that actively divert carbon and nitrogen toward protective pathways. When these stresses co-occur, allocation trade-offs between growth, maintenance, reproduction, and defense become more acute. However, most current growth models parameterize photosynthesis, respiration, and nitrogen assimilation under optimal or single-stress conditions, assuming stable relationships between environmental drivers (temperature, *CO*_2_, water availability) and metabolic rates. Under agroecological or low-input conditions, such assumptions may no longer hold, as interacting stresses alter both the availability of resources and the priorities governing their allocation. For example, plants grown under low nitrogen availability may exhibit reduced photosynthetic capacity not only due to limited Rubisco content, but also because nitrogen is preferentially allocated to inducible defenses in anticipation of biotic stress, a dynamic reallocation that is not captured by standard nitrogen-photosynthesis relationships. To accurately represent plant performance in complex agroecological systems, models must therefore move beyond fixed parameterizations toward dynamic, mechanistic representations of resource allocation that explicitly account for the metabolic costs of defense and the context-dependent trade-offs between growth, defense, and maintenance.

### 4.3 Modeling strategies for representing combined stresses and defense costs

Several modeling strategies can be employed to represent combined stress effects. A first approach involves introducing interaction terms or nonlinear response surfaces in which the combined effect of two stresses (e.g., drought and pathogen infection) is represented not as the sum of individual effects, but through multiplicative terms or polynomial functions that capture synergistic or antagonistic interactions (Piggott et al., 2015; Webber et al., 2022). For instance, photosynthesis under combined heat and water stress might decline more rapidly than the additive prediction, requiring nonlinear response surfaces (Li et al., 2025). A second approach implements stress priority or dominance rules, where the most limiting factor at a given time determines allocation priorities. For example, when water availability drops below a critical threshold, defense responses to biotic stress might be suppressed in favor of drought survival mechanisms, reflecting a hierarchical control where acute threats override chronic ones. A third strategy adopts modular or hierarchical control architectures in which different stress signals are integrated through hormone-mediated regulatory networks at multiple organizational levels (tissue, organ, whole plant). In this framework, local stress perception at the tissue level triggers modular responses (e.g., defense compound synthesis at infection sites), while systemic signals coordinate whole-plant adjustments (e.g., redistribution of nitrogen away from growth toward defense). Such hierarchical models can accommodate both autonomous local responses and coordinated system-level prioritization, thereby better reflecting the biological reality of stress perception and response integration.

From a modeling perspective, growth and defense regulation cannot be represented as a single, uniform control acting at the scale of the whole plant. Instead, stress responses involve organ-specific adjustments that must be represented explicitly, while remaining coordinated through shared plant-level resource pools, notably carbon and nitrogen. At the same time, the level of biological detail introduced must remain compatible with PBM and FSPM frameworks. Rather than explicitly modeling detailed gene or hormonal networks, which would substantially increase complexity and data requirements, stress effects can be introduced through simplified regulatory terms acting at the organ or whole-plant level. These terms may modulate source capacities (e.g. photosynthesis or nutrient uptake), allocation priorities, sink strengths, or maintenance demands, while coordination across organs is ensured through common resource pools.

Stress regulation is also inherently dynamic. Delays, feedback loops, and hysteresis are inherent to hormonal signaling and play a critical role in shaping plant responses under fluctuating or repeated stress exposure. Accounting for such temporal dynamics would improve the realism of growth simulations and strengthen the capacity of models to represent growth-defense trade-off in complex environments. However, incorporating these dynamics remains challenging, as the underlying mechanisms, timescales, and interactions between regulatory pathways are still only partially understood and may vary across genotypes, developmental stages, and environmental contexts, making their generic parameterization difficult.

Proxy variables may offer practical approximations for model parameterization. For instance, the total respiration rate can serve as a proxy for overall metabolic activity including defense, while changes in tissue C:N ratios may indicate shifts between structural growth and protein-based defense. Gene expression levels of defense-related pathways (e.g., phenylpropanoid biosynthesis genes) or hormone concentrations (e.g., SA, JA) could also provide real-time indicators of defense activation intensity, enabling dynamic adjustment of allocation coefficients (Bari and Jones, 2009; He et al., 2022). Near-infrared spectroscopy (NIRS) profiles, which correlate with concentrations of secondary metabolites, offer a non-destructive means of estimating defense investment (Calzone et al., 2021; Nantongo et al., 2021). By incorporating such proxies into models, it becomes possible to represent the magnitude of growth-defense trade-off without requiring exhaustive biochemical characterization of every defense pathway.

Within this framework, these proxy measurements provide a practical basis for defining defense coefficients as conceptual variables rather than fully specified variables, reflecting genotype-dependent differences in defense strategies, dynamic responses to stress, or a combination of both. From a modeling perspective, it is more appropriate to represent these coefficients as continuous variables modulating source-sink relationships and organ-level allocation rules, rather than as binary stress indicators, even if their mathematical formulation and parameterization remain to be defined. However, from a practical standpoint, an intermediate step based on categorical or discretized variables (e.g. low, moderate or high defense activation states) could provide a good basis, facilitating model calibration and data integration before embracing fully continuous formulations.

The integration of molecular information offers promising perspectives to inform such defense coefficients. Previous work has shown that combining advanced omics approaches with plant–microbe interactions and tailored nutrient management can improve plant resilience to multiple stresses (Khan et al., 2025). Crosstalk between phytohormone signaling, oxidative stress regulation, and transcription factor networks appears central to cross-tolerance under combined stress conditions. By measuring molecular responses at the organ level, it becomes possible to take a snapshot of the plant biochemical response at the tissue level that will be translated into a physiological response and to a final trait adaptation. Thus, recent modeling approaches aim to predict the phenotypic traits of a plant based on molecular data combining different origins (Bleker et al., 2024). Building on this knowledge, it could be possible to define defense coefficients based on the presence or expression of certain genes or metabolites, and integrate them into models via allocation as presented by Baenziger et al. (2004) for height reduction genes.

## 5 A conceptual model of wheat growth adapted to a fluctuating environment

In this context, our objective is to propose a conceptual framework between PBMs and FSPMs, based on current knowledge, for wheat growth that explicitly incorporates the trade-off between growth and defense. This framework is intended as a conceptual basis for future model development rather than a fully parameterized implementation. Such a model could not only provide a better understanding of resource allocation under biotic and abiotic stress conditions, but also lay the foundations for improved explanatory and predictive models in more complex agroecological systems. Ultimately, this type of model could be used to assess the effectiveness of agronomic levers in greater detail, particularly those based on exogenous applications of phytohormones (Swain et al., 2023). Whether biocontrol agents, natural defense stimulators or biostimulants promoting root growth or nutrient uptake, these strategies aim to modulate the plant’s physiological balance (Figueroa-Macías et al., 2021). Understanding how these products influence resource allocation, and at what cost, is crucial. A model integrating the growth-defense trade-off could thus become a decision-making tool to optimize the positioning of these biosolutions in crop management strategies.

### 5.1 Model description

**Figure 3:**
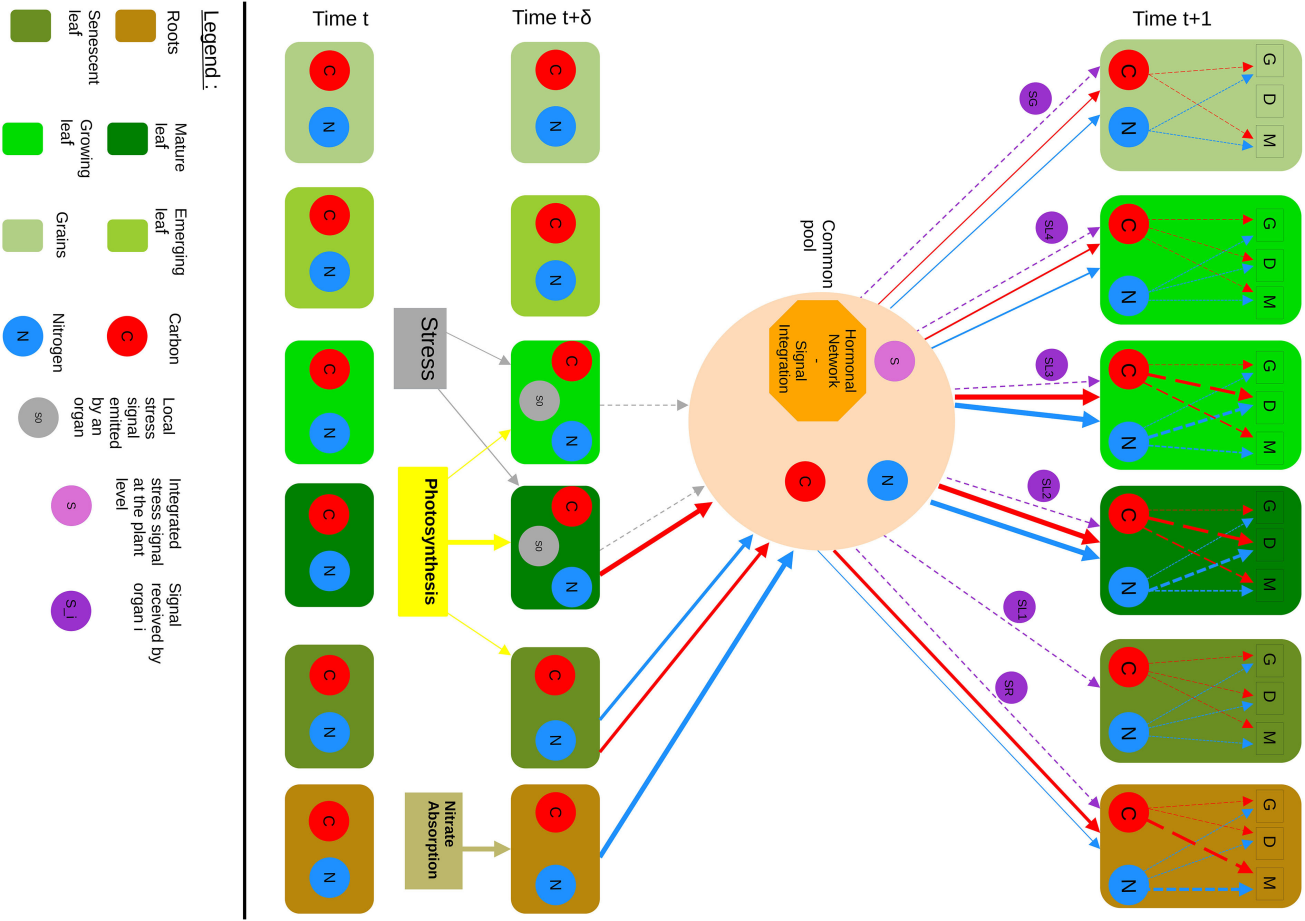
Conceptual model of resource allocation in wheat under stress. Each box represents a functional organ or plant compartment (roots, senescent leaf, mature leaf, growing leaf, emerging leaf, and grains). Red arrows indicate carbon fluxes, blue arrows indicate nitrogen fluxes. Grey arrows represent local stress signals emitted by organs, while purple arrows indicate integrated regulatory signals distributed to individual organs. The model operates in discrete time steps. At time *t*: Mature and growing leaves assimilate carbon through photosynthesis, while roots absorb nitrogen through nitrate uptake. Stressed organs generate local stress signals (S0), reflecting their physiological state. At time *t* + *δ*: Carbon and nitrogen accumulated in each organ are transferred to a common pool. Simultaneously, stress signals from affected organs are transmitted to a central integration unit, which aggregates information about the overall plant physiological state. At time *t* + 1: The central integration unit redistributes carbon and nitrogen from the common pool to individual organs according to their relative demand and priority status. Allocation is modulated by an integrated hormonal regulation module, which adjusts source-sink dynamics in response to stress. Each organ receives a regulatory signal reflecting the global plant status. Within organs, resources are partitioned among growth (G), defense (D), and maintenance (M) processes according to the received signal.

**Figure 3** summarizes the conceptual structure of the model, highlighting organ compartments, carbon and nitrogen flows (red and blue arrows respectively), stress signals (grey arrows), and information exchanges (purple arrows). The model is based on a discrete-time compartmental representation at the organ-plant scale. Carbon and nitrogen assimilated by the plant are assumed to be stored in a common pool and then redistributed among organs according to their relative demands. At each time step, certain constraints are applied: the total amount of carbon and nitrogen allocated cannot exceed available stocks, and unused resources can remain in the common pool or be returned to it. Each organ is represented as a compartment whose internal resources are divided between three main processes: maintenance, defense, and growth.

Time is represented discretely, typically at a daily scale. At each time *t*, the plant is in a particular physiological state. The interval between *t* and *t* +Δ corresponds to rapid processes such as signal perception and integration, while allocation decisions affect organs at *t* + 1. This structure enables the implicit introduction of delays between stress perception and resource reallocation, while assuming that, at the scale of an herbaceous plant such as wheat, transport times of information and assimilates between organs are negligible relative to the chosen time step. The step resolution could be adapted depending on the model and the data available.

At each time *t*, carbon assimilation is computed at the leaf level using established formulations based on the Farquhar et al. (1980) photosynthesis model coupled with BWB stomatal conductance models (Ball et al., 1987), providing a direct link between environmental conditions, plant physiological state, and carbon fluxes. Nitrogen uptake is represented following the formulation used in the CN-Wheat (Barillot et al., 2016), combining the HATS and LATS transport systems and their regulation by root carbon status. These formulations are compiled in the **Supplementary Material**.

### 5.2 Stress signal integration

Each organ exposed to biotic or abiotic stress emits a local stress signal (S0), representing its physiological perturbation or its local stress state (**Figure 3**). These dimensionless stress intensity values could be assessed in different ways, potentially derived from measurable proxies such as phytohormone concentrations, transcriptional markers of stress perception, or NIRS profiles (Dietz and Vogelsang, 2024; Cooper et al., 2025). Rather than assuming a biological “central controller”, the signal integration unit represents an abstract aggregation of these signals at the plant level. It summarizes the information about the local stress signals and the overall plant status into a limited set of variables that adjust allocation priorities. This representation allows coordination of allocation decisions without explicitly simulating the underlying molecular networks.

These defense coefficients would function as dynamic state variables rather than static parameters, evolving in response to stress intensity, duration, and the plant’s physiological state. Specifically, a defense coefficient (*D*) could be calculated as a normalized function of molecular indicators:


D=f(gene expression, hormone concentration, metabolite levels)

where f represents an empirical or semi-mechanistic relationship calibrated from experimental data. The coefficient would scale with stress intensity following a dose-response relationship (e.g., sigmoidal or hyperbolic functions), reflecting the saturation of defense responses at high stress levels, and would exhibit temporal dynamics with both induction (increase during stress exposure) and relaxation (gradual decline after stress removal) phases. Genotypic variation would be captured through cultivar-specific parameterizations of the f relationship, allowing representation of differential defense capacities among varieties.

These defense coefficients would interact with existing model components through multiple pathways. In respiration modules, the defense coefficient would modulate maintenance respiration costs:


$$R_{maintenance}=R_{basal}+D\times C_{defense}$$

where $C_{defense}$ represents the per-unit respiratory cost of defense activation. This makes defense costs explicit rather than lumping them into a generic maintenance term. In source-sink allocation frameworks, defense would compete with growth as an explicit sink, with sink strength proportional to the defense coefficient:


$$Sink_{defense}=D\times Organ_{mass}$$

meaning that organs under stress become active consumers of resources for defense purposes. Quantifying the expression of certain genes could thus serve as a first approach to estimating the costs associated with defense, with the possibility of adapting the response depending on the cultivar.

While finer levels (e.g., stomatal or cellular processes) are not represented in this framework, they could be incorporated in future developments. The proposed integration should therefore be understood as a simplified representation rather than a fully mechanistic approach, favoring the use of intermediate information—such as stress knowledge maps or network syntheses derived from biostatistics—over explicit modeling of complete molecular pathways.

### 5.3 Resources allocation rules

As illustrated in **Figure 3**, at each time step *t* + 1, carbon and nitrogen available in the common pool are redistributed among organs according to their relative demands reflecting their developmental stage and functional role. Allocation among organs follows a source–sink logic, regulated by an integration unit that aggregates hormonal and stress-related signals to modulate inter-organ flows. Besides resource allocation, the integration unit supplies each organ with information on the plant’s overall physiological state and a set of allocation directives, defining the relative priority of maintenance, defense, and growth processes. These directives may also include functional constraints, such as the downregulation of source activities (e.g. photosynthesis or nutrient uptake) or the transition toward senescence under prolonged or severe stress. Organ demand and priority levels may vary over phenological time (e.g. actively growing leaves during vegetative stages, grains during reproductive stages), but are assumed to remain constant within a given time step. In addition, organ-specific stress signals modulate effective demand: under stress, resources may be preferentially redirected toward affected organs to sustain maintenance or defense, while allocation to less critical or non-stressed organs is reduced. Inter-organ allocation thus emerges from the combined weighting of organ demand by priority status and stress modulation, under the constraint of global carbon and nitrogen availability. Total allocation is bounded by the resources available in the common pool. Within each organ, carbon and nitrogen are divided among three competing functions: maintenance, defense, and growth. This partitioning could follow a hierarchical and constrained allocation scheme. At each time step, maintenance demand is satisfied first. If available resources are insufficient to fill maintenance requirements, no allocation to defense or growth occurs. Persistent failure to fill maintenance demand could lead to degradation or senescence in future model extensions. Resources remaining after maintenance are then allocated to defense or growth processes according to the local signal perceived by the organ. Under low stress intensity, allocation is preferentially directed toward growth, whereas increasing stress progressively directs allocation toward defense. Unused resources may then be returned to the common pool.

## 6 Modeling implementation: limitations and challenges

### 6.1 Model limitations

The assumption of a centralized and instantaneous integration unit, as shown in **Figure 3**, represents a simplification of plant functioning. In real wheat plants, source–sink regulation emerges from a combination of local feedbacks, spatial heterogeneity, and temporal delays. For example, carbohydrate accumulation in leaves can locally downregulate photosynthesis, root nitrogen uptake responds to local carbon availability with time lags, and stress responses may initially remain organ-specific before triggering systemic signals. At this stage, these local controls and time lags are not explicitly represented, but some consequences of local feedbacks are implicitly captured (e.g. allocation outcomes affect organ growth and activity, which in turn modify organ demand and contribution at subsequent time steps). Moreover, the discrete-time formulation introduces an explicit separation between rapid processes of stress perception and signal integration, occurring within a time step, and the slower effects of resource reallocation, which become effective at the next time step. The integration unit should therefore not be interpreted as a biological entity, but as an abstract coordination unit that aggregates the effects of regulatory processes operating at multiple levels. This assumption improves conceptual clarity and model tractability, but inevitably neglects local dynamics and short-term feedbacks that influence growth and resource allocation in wheat. Explicit consideration of these processes would require finer spatial and temporal resolution, leading to increased computational complexity and data requirements.

Although this work focuses on establishing the conceptual structure of the model, this conceptual framework is intended to serve as the foundation for a future quantitative implementation. As a first step, the conceptual structure presented here is designed as a discrete-time compartmental model. In such a formulation, each organ is treated as a compartment whose carbon and nitrogen stocks are updated at successive time steps according to the incoming flows and allocation rules. In future developments, this discrete representation could be extended to a system of ordinary differential equations to describe continuous carbon and nitrogen dynamics, thereby offering a more mechanistic and physiologically detailed formulation. Moreover, several well-established equations describing photosynthesis, stomatal conductance, respiration, and nitrogen uptake are compiled in the **Supplementary Material**. These equations offer ready-to-use formulations that can be directly integrated into future model developments.

### 6.2 From conceptual framework to quantitative implementation

Key considerations for model refinement will include several major elements. One element is the identifiability of parameters. Can parameters be reliably estimated from available data, or does added complexity lead to equifinality? Does increased complexity improve model performance on independent validation datasets, or do simpler models suffice? Are the measurements required to parameterize complex mechanisms (e.g., tissue-specific hormone concentrations, gene expression time series) feasible at relevant spatial and temporal scales?

To operationalize this framework, we propose different steps. The first step will be to implement a parsimonious model extension within existing FSPM frameworks (e.g., CN-Wheat or ADEL-Wheat). This requires the addition of: (i) dynamic defense coefficients as state variables responding to stress signals, (ii) explicit defense sinks competing with growth in the allocation routine, and (iii) stress-modulated respiration costs. This minimal extension would maintain computational tractability while introducing the core trade-off mechanism. During the second step, we would progressively add complexity based on model performance and data availability, potentially incorporating hormonal state variables (SA, JA, ABA concentrations at organ level), stress interaction terms (e.g., drought × pathogen response surfaces), and cultivar-specific defense parameterizations.

Model validation and potential falsification would proceed through multi-level experimental datasets comparing model outputs against independent observations. At the physiological level, validation would test whether the model correctly predicts growth reductions under single and combined stresses: experiments manipulating pathogen inoculation and water availability in controlled conditions would provide time-series data on biomass accumulation, leaf area development, and grain yield that could be compared against simulated trajectories. A key falsifiable prediction is that combined stresses should produce non-additive effects on growth, with the magnitude and direction (synergistic or antagonistic) depending on stress timing and intensity as captured by the interaction terms. At the metabolic level, validation would assess whether simulated resource allocation patterns match observed C and N partitioning: measurements of tissue C:N ratios, soluble sugar concentrations, and defense metabolite levels (e.g., total phenolics) under stress would test whether the model correctly represents the diversion of resources to defense (Rubia et al., 2025). At the molecular level, predicted defense activation (as reflected in defense coefficient values) could be validated against measured hormone concentrations or defense gene expression, testing whether the reduced-order representation captures the essential dynamics of the underlying regulatory networks. Critically, validation should emphasize out-of-sample prediction: the model should be parameterized using data from specific cultivar × stress combinations, then tested on independent cultivars, different stress intensities, or novel stress combinations not used in calibration. Failure to predict these independent scenarios would indicate either insufficient model complexity or incorrect mechanistic assumptions, guiding iterative model refinement.

The model will be evaluated based on its ability to better reproduce observed growth and allocation patterns, compared with reference growth models, using independent datasets covering different environments and different stresses, either single or combined. More specifically, we will verify whether explicitly taking into account defense costs and stress-regulated allocation rules leads to a more realistic representation of biomass distribution. From a practical perspective, model validation should rely on multi-level experimental datasets, comparing predictions against independent observations across physiological, metabolic, and molecular scales. The calibration of such a framework would rely on datasets combining classical growth measurements (e.g. dry mass, leaf area, nitrogen content) with organ-level information on defense activation, such as gene expression data, relative concentrations of structural or secondary compounds, or non-destructive spectral proxies derived from NIRS reflecting changes in biochemical composition and physiological status. These measurements can be complemented by readily observable indicators of plant physiological status, including traits related to photosynthetic capacity (e.g. chlorophyll content), stress-induced tissue degradation (e.g. senescence or disease severity), and changes in respiratory demand associated with maintenance and repair processes. Together with basic environmental descriptors of abiotic and biotic stress intensity, such data provide indirect but informative constraints on organ status and whole-plant resource allocation. Recent studies combining metabolomic, transcriptomic, and growth measurements under mild stress conditions illustrate how such datasets can be used to characterize plant physiological states and infer changes in resource allocation, providing a realistic basis for model validation or falsification (Olas et al., 2021).

The model should then be evaluated on independent datasets (e.g., novel cultivars, stress intensities, or combinations) to assess its generalization capacity and guide iterative refinement. Failure to predict these scenarios would indicate insufficient complexity or incorrect mechanistic assumptions, guiding iterative refinement.

### 6.3 Modeling challenges: balancing complexity and parsimony

A central challenge in developing such models lies in striking the right balance between mechanistic realism and practical tractability. This question highlights a fundamental tension in biological modeling, namely the trade-off between mechanistic realism and practical tractability. More detailed models that explicitly represent defense metabolite synthesis pathways, hormonal crosstalk networks, and gene regulatory circuits offer greater explanatory power and can potentially capture emergent behaviors that simplified models miss. However, such complexity comes at the cost of increased parameter uncertainty, higher computational demands, and greater data requirements for calibration, thus potentially limiting applicability in operational contexts. Conversely, parsimonious models with reduced-order representations (e.g., defense coefficients derived from aggregated molecular indicators) sacrifice mechanistic detail but gain in transparency, computational efficiency, and ease of parameterization. The appropriate level of detail should be determined by the intended model application and the principle of “minimum sufficient complexity”: the model should be complex enough to capture the phenomena of interest (here, growth-defense trade-offs under combined stresses) but no more complex than necessary given available data and computational resources. Practical criteria for this decision include: (i) identifiability: can model parameters be reliably estimated from available data, or does adding complexity lead to equifinality where multiple parameter sets produce similar outputs? (ii) predictive gain: does increased complexity substantially improve model performance on independent validation data, or do simpler models perform comparably? (iii) data availability: are the measurements required to parameterize complex mechanisms (e.g., tissue-specific hormone concentrations, gene expression time series) feasible at the spatial and temporal scales of interest? Answering these questions requires systematic model comparison and validation against empirical data.

At this stage, the model does not explicitly simulate organ mortality or the dynamic processes of stress-induced senescence. However, sustained or severe stress may lead to functional downregulation or loss of activity at the organ level, represented implicitly through allocation directives and reduced source or sink capacities. Incorporating explicit thresholds of irreversible damage or stress-driven senescence will constitute a possible and necessary extension of the model. Furthermore, soil processes and water dynamics are not explicitly modeled, and both soil nitrogen availability and water status are assumed to be non-limiting. In future developments, these assumptions could be relaxed to incorporate soil–plant interactions, allowing nitrogen availability and water status to act as abiotic stress factors.

Choosing the scale of a model is a critical decision, as it directly conditions its explanatory power, complexity, and compatibility with available data. The framework proposed here is primarily formulated at the plant and organ scales, which allows explicit representation of major functional trade-offs between growth, defense, and maintenance through a source–sink perspective, while maintaining a level of complexity compatible with realistic datasets. This choice also enables local organ-level processes to influence whole-plant behavior through demand, priority, and stress modulation, thereby articulating multiple organizational levels. While our framework focuses on organ- and plant-level resource allocation, growth-defense trade-offs also manifest at the canopy scale. For example, defense prioritization in specific leaves may reduce carbon investment in growth, accelerate tissue turnover, and alter light interception, ultimately reshaping canopy structure and biomass production. Future developments could extend the model to canopy-level dynamics, integrating light competition, microclimate effects, and whole-plant architectural responses to stress.

A common challenge in integrative plant models is the risk of over-parameterization. Here, this risk is mitigated by deliberately simplifying allocation rules and constraining them using biologically interpretable parameters (e.g. organ-specific demand and priority coefficients, maintenance requirements). Allocation is governed by explicit priority structures, stress-dependent modulation, and resource constraints, rather than by fully continuous and unconstrained functions. Such strategies, inspired by classical plant modeling approaches (e.g. Thornley and Cannell (2000)), enhance parameter identifiability and model robustness when confronted with realistic experimental datasets.

## 7 Conclusions and outlook

Over the past decades, plant growth models have evolved substantially, progressively incorporating physiological, environmental, and, more recently, genetic processes. However, they still face important limitations when it comes to representing physiological trade-offs, particularly the balance between growth and defense in complex environments where multiple biotic and abiotic stresses interact. A plant’s capacity to allocate resources among vital functions depends on dynamic regulatory mechanisms, which remain insufficiently captured by current models. To address this gap, we have proposed a conceptual framework for wheat growth that explicitly integrates this trade-off, drawing on hormonal and gene regulation modulated by the plant’s environment and physiological state. Such an approach offers promising avenues to enhance both the explanatory power and predictive robustness of modeling tools in complex agroecological contexts. In particular, such models could be used to support experimental design for solutions based on plant physiology such as plant resistance inducers or plant growth biostimulants.

Beyond wheat, such methodologies could be extended to other species and contribute to the design of cropping systems that reconcile productivity, resilience, and resource efficiency. The rapid expansion of high-throughput phenotyping and other data acquisition technologies creates new opportunities for intensive model validation and parameterization. The success of such endeavors depends on close collaboration between experimental research and modeling, with experimental designs explicitly informed by model requirements (e.g., measuring specific variables at time scales relevant to model processes) and models iteratively refined based on discrepancies between predictions and observations.
