# Supplementary material for: Wheat growth model capturing growth-defense trade-off
Source: Front Plant Sci. 2026 Mar 12;17:1763868. doi: 10.3389/fpls.2026.1763868 (PMC13017885; doi:10.3389/fpls.2026.1763868)
Supplement: Supplementary file 1 [file Table1.docx]

Supplementary Material

Some useful mathematical equations underlying growth models

The table below presents physiological equations taken from published models that are commonly used to describe carbon assimilation, stomatal conductance, respiration, and nitrogen uptake.These equations, drawn from well-established references, provide a foundation for guiding the construction of our own mechanistic model. They are organized according to the physiological function they represent and are accompanied by their description, and bibliographic source.

**Table S1 :** Physiological Equations Extracted from Published Plant Models

| **Equation** | **Description** | **Reference** |
| --- | --- | --- |
| **Photosynthetic rate** | | |
| $A_{g}\text{=}min\left( A_{c},A_{j},A_{p} \right)$ | Gross photosynthetic rate | Farquhar et al. (1980) |
| $A_{n}\text{=}A_{g}-R_{d}^{FCB}$ | Net photosynthetic rate | - |
| $A_{j}\text{=}\frac{J*\left( C-\Gamma^{\bullet} \right)}{4C\text{+}8\Gamma^{\bullet}}$ | RuBP regeneration-limited carboxylation rate | - |
| $A_{c}\text{=}\frac{V_{Cmax}*\left( C-\Gamma^{\bullet} \right)}{C\text{+}K_{c}*\left( 1\text{+}\frac{O}{K_{o}} \right)}$ | RubisCO-limited carboxylation rate | - |
| $A_{p}\text{=}\left( 1-\frac{\Gamma^{\bullet}}{C} \right)*\left( 3TPU\text{+}V_{o} \right)$ | TPU-limited carboxylation rate | - |
| **Stomatal conductance (BWB model)** | | |
| $g_{sw}\text{=}g_{0}\text{+}k\frac{A_{g}*h_{s}}{C_{s}}$ | Stomatal conductance to water | Ball et al. (1987) |
| $C_{s}\text{=}C_{a}-\frac{A_{n}*1,37}{g_{b}}$ | ${CO}_{2}$ concentration at organ surface | Barillot et al. (2016) |
| **Respiration** | | |
| $R_{organ}^{growth}\left( t \right)\text{=}\frac{\left( 1-Y_{g} \right)*G\left( C,N \right)\left( t \right)}{Y_{g}}$ | Local growth respiration rate of an organ | Thornley and Cannell (2000) |
| $R_{organ}^{residual}\left( t \right)\text{=}\frac{k_{m,max}*\left[ C_{organ} \right]\left( t \right)*N_{organ}^{total}\left( t \right)}{K_{m}\text{+}\left[ C_{organ} \right]\left( t \right)}$ | Residual respiration rate of an organ | Barillot et al (2016) |
| **Nitrate uptake** | | |
| $HATS\left( t \right)\text{=}\frac{\left[ N_{soil}^{nit} \right]\left( t \right)*{Vmax}_{roots}^{N,nitHATS}}{\left[ N_{soil}^{nit} \right]\left( t \right)\text{+}K_{roots}^{N,nitHATS}}$ | Calculation of the transport system HATS (high-affinity system) | Barillot el al. (2016) |
| $LATS\left( t \right)\text{=}K_{roots}^{N,nitLATS}*\left[ N_{soil}^{nit} \right]\left( t \right)$ | Calculation of the transport system LATS (low-affinity system) | - |
| ${Influx}_{roots}^{N,nit}\left( t \right)\text{=}\left( HATS\left( t \right)\text{+}LATS\left( t \right) \right)*M_{roots}^{struct}\left( t \right)$ | Rate of nitrate influx | - |
| ${Uptake}_{roots}^{N,nit}\left( t \right)\text{=}{Influx}_{roots}^{N,nit}\left( t \right)*r_{influx:netuptake}*f_{CHO}\left( t \right)$ | Net nitrate uptake rate | - |

**Table S2 :** Symbols, description and units of the parameters

| **Symbol** | **Description** | **Unit** |
| --- | --- | --- |
| **Photosynthesis** | | |
| $R_{d}^{FCB}$ | Mitochondrial respiration rate in light | $\mu molm^{-2}s^{-1}$ |
| J | Electron transport rate | $\mu mol$ e- $m^{-2}s^{-1}$ |
| C | Intercellular ${CO}_{2}$ concentration | $\mu mol{CO}_{2}{mol}^{-1}$ |
| $\Gamma^{\bullet}$ | ${CO}_{2}$ compensation point in absence of dark respiration | $\mu mol{CO}_{2}{mol}^{-1}$ |
| $V_{Cmax}$ | Maximum rate of RubisCO carboxylation | $\mu mol{CO}_{2}m^{-2}s^{-1}$ |
| $K_{c}$ | Affinity constant of RubisCO for C | $\mu mol{mol}^{-1}$ |
| O | Intercellular $O_{2}$ concentration | $\mu mol{mol}^{-1}$ |
| $K_{o}$ | Affinity constant of RubisCO for O | $\mu mol{mol}^{-1}$ |
| $V_{o}$ | Rate of RubisCO oxygenation | $\mu molO_{2}m^{-2}s^{-1}$ |
| TPU | Triose phosphate utilisation rate | $\mu mol{CO}_{2}m^{-2}s^{-1}$ |
| **Stomatal conductance** | | |
| $g_{0}$ | Minimum $g_{sw}$ measured in the dark | $mol{mol}^{-1}$ |
| k | Composite sensitivity of stomatal conductance to assimilation | *Dimensionless* |
| $h_{s}$ | Relative humidity at the leaf surface | *Dimensionless* |
| $C_{a}$ | Ambient ${CO}_{2}$ concentration | $\mu mol{CO}_{2}{mol}^{-1}$ |
| $g_{b}$ | Boundary layer conductance to water | $molm^{-2}s^{-1}$ |
|  |  |  |
| **Respiration** | | |
| $Y_{g}$ | Growth yield | *Units of C appearing in product M per unit of C substrate utilized for growth* |
| G(C,N) | Gross growth rate | *Units of C in plant tissue per day* |
| $\left[ C_{organ} \right]$ | C substrate concentration | $\mu molg^{-1}$ |
| $K_{m}$ | Michaelis-Menten constant affinity | $\mu molg^{-1}$ |
| $k_{m,max}$ | Maximum value of the maintenance constant when $\left[ C_{organ} \right]$ is much greater than $K_{m}$ | $\mu mol$ *of C substrate respired per* $\mu molNs^{-1}$ |
| $N_{organ}^{total}$ | Total amount of organic N contained in the organ | $\mu molN$ |
|  |  |  |
| **Nitrate uptake** | | |
| $\left[ N_{soil}^{nit} \right]$ | Nitrate concentration soil | $\mu molm^{-3}$ |
| ${Vmax}_{roots}^{N,nitHATS}$ | Maximum rate of nitrate uptake for HATS | $\mu molNg^{-1}s^{-1}$ |
| $K_{roots}^{N,nitHATS}$ | Affinity coefficient of nitrate uptake for HATS | $\mu molNg^{-1}$ |
| $K_{roots}^{N,nitLATS}$ | Rate of nitrate uptake for LATS | $m^{3}g^{-1}s^{-1}$ |
| $r_{influx:netuptake}$ | Ratio of nitrate influx on net uptake | *Dimensionless* |
| $f_{CHO}$ | Regulation of nitrate uptake by root carbohydrate concentration | *Dimensionless* |
| $M_{roots}^{struct}$ | Root structural dry mass | *g* |
